# *IGFBP5* Promotes Atherosclerosis in APOE^−/−^ Mice Through Phenotypic Transformation of VSMCs

**DOI:** 10.3390/cimb47070555

**Published:** 2025-07-17

**Authors:** Aoqi Xiang, Hua Guan, Peihong Su, Lusha Zhang, Xiaochang Chen, Qi Yu

**Affiliations:** Shaanxi Key Laboratory of Ischaemic Cardiovascular Diseases & Institute of Basic and Translational Medicine, Xi’an Medical University, Xi’an 710021, China; xiangaoqi_jzs@xiyi.edu.cn (A.X.); guanhua@stu.xjtu.edu.cn (H.G.); suph@xiyi.edu.cn (P.S.); zhanglusha2022@xiyi.edu.cn (L.Z.)

**Keywords:** atherosclerosis, *IGFBP5*, ApoE^−/−^ mice, VSMCs, phenotypic transformation

## Abstract

Atherosclerosis constitutes a pathological process underlying cardiovascular diseases. There is growing evidence that *IGFBP5* is a causative factor, although the conclusions of different studies are inconsistent. The present study aims to confirm the role and mechanism of *IGFBP5* in atherosclerosis. The expression of *IGFBP5* was induced in the skeletal muscle of male ApoE^−/−^ mice, an atherosclerosis model, using adeno-associated virus, resulting in elevated circulating *IGFBP5* levels. Changes in blood lipids were detected, and pathological changes in the aorta were observed. Analysis of *IGFBP5* function using RNA sequencing and validation were performed in a mouse aortic smooth muscle cell line. The results demonstrated that *IGFBP5* overexpression exacerbated the development of aortic lesions in this murine models without any discernible alterations in lipid profile parameters; the arterial transcriptomic landscape revealed that heightened *IGFBP5* levels predominantly influenced pathways governing smooth muscle cell proliferation and motility. In vitro experimentation corroborated these findings, showcasing the stimulatory effect of *IGFBP5* on VSMC (vascular smooth muscle cell) proliferation and migration, provoking a transition toward a proliferative phenotype. *IGFBP5* promotes atherosclerosis in ApoE^−/−^ mice through the phenotypic transformation of VSMCs. This finding suggests that *IGFBP5* has the potential to serve as an indicator of atherosclerosis diagnosis and a target for therapeutic interventions in the future.

## 1. Introduction

Cardiovascular disease is an increasing threat to human health and longevity, imposing an overwhelming strain upon healthcare systems worldwide [1]. At the epicenter of this epidemic lies atherosclerosis—the cardinal pathophysiological underpinning propelling cardiovascular disorders into epidemic proportions [2]. The genesis of atherosclerotic plaques is a multi-faceted process, influenced predominantly by four principal determinants: ① Dysregulation of Blood Lipids: Elevated lipid profiles, particularly soaring levels of low-density lipoprotein (LDL), often referred to as “bad cholesterol,” represent the primary lipid reservoir within nascent plaques [3]. ② Endothelial Dysfunction: Damage to the arterial lining, or endothelium, constitutes a pivotal initial event in the cascade leading to plaque formation. Endothelial disruption fosters an environment conducive to inflammation and thrombosis [4]. ③ Inflammatory Cells and Macrophage Activity: Activated immune cells, most notably macrophages, infiltrate the subendothelial space where they engulf excess lipids, transforming into foam cells—a hallmark feature of early-stage atherosclerosis [5]. ④ Phenotypic Transformation of VSMCs: Plaque composition includes a considerable contingent of cells originating from VSMCs, which have undergone dramatic phenotypic shifts [6]. These cells may assume diverse roles akin to macrophages, mesenchymal stem cells, or osteoblastic lineages, culminating in the assembly of plaques with intricate cellular heterogeneity [3,7].

Insulin-like Growth Factor Binding Protein 5 (*IGFBP5*), a constituent of the IGF binding protein clan, is expressed in a variety of different tissues throughout the body including muscle, bone, lung, etc., and its secretion is mediated by an N-terminal signal peptide [8]. Circulating *IGFBP5* is found both in ternary complexes, with IGFs and an acid-labile subunit, and binary complexes together with IGFs [9]. *IGFBP5* exerts its function in part by regulating the bioavailability of IGFs. In addition, *IGFBP5* also has activities that are independent of IGFs and can even enter the cell nucleus to exert its effects [8]. Its mechanism of action is relatively complex. Studies have found that *IGFBP5* modulates lipid metabolism and insulin sensitivity in non-alcoholic fatty liver disease [10] and suppresses intramyocellular lipids deposition [11]. *IGFBP5* also stimulates VSMC migration [12] and human intestinal smooth muscle cell growth [13] in an IGF-independent manner, indicating that *IGFBP5* plays a significant role in regulating lipid metabolism and smooth muscle function.

A cross-sectional study from Germany included total of 95 nondiabetic male patients with coronary heart disease (CHD) and 92 controls who were below the age of 60 years and matched by age, body mass index (BMI), and smoking habits. The presence of CHD had significant positive associations with *IGFBP5*, and these associations were independent of other traditional risk factors [14]. There have also been recent studies on the relationship between *IGFBP5* and cardiovascular disease. For example, Wang et al. found that the serum *IGFBP5* protein level in patients with deep vein thrombosis was significantly increased [15]. Zhu et al. found that the serum *IGFBP5* level in patients with acute myocardial infarction was significantly increased and positively correlated with the risk of short-term major adverse cardiovascular events [16]. Notably, *IGFBP5* expression is amplified within atherosclerotic lesions, wherein copious quantities of the protein are observed in intimate association with the extracellular matrix enveloping these plaques [17,18]. Furthermore, *IGFBP5* promotes VSMC senescence, and elevated *IGFBP5* in human atherosclerotic plaques is accompanied by increased chronic inflammation [19]. Yet, a discordant narrative emerges. Xu et al. found that *IGFBP5* possesses anti-inflammatory properties vis-a-vis endothelial cells, with its expression conspicuously diminished within human atherosclerotic plaques. These findings suggested that *IGFBP5* inhibits inflammation, a major precondition for atherosclerosis [20]. Thus, the precise function of *IGFBP5* in atherosclerosis remains shrouded in ambiguity, lacking definitive elucidation or targeted examination. The mechanism by which it operates in this context is yet to be fully unraveled, underscoring a critical gap in our understanding.

In this study, we selected ApoE^−/−^ mice as the main research subjects, as they are the most commonly used animal model of spontaneous atherosclerosis and exhibit a significant increase in plasma cholesterol. We found that *IGFBP5* is a catalyst for the aggravation of atherosclerotic plaque accumulation in ApoE^−/−^ mice. Using transcriptomic sequencing, we embarked on deciphering the directional sway exerted by *IGFBP5*. Our findings unveiled a notable diminution in the expression profiles of markers characteristic of the contractile phenotype of VSMCs. Strikingly, this reduction was not paralleled by a marked elevation in the transcriptional signatures of genes emblematic of macrophage-like phenotypes.

## 2. Materials and Methods

### 2.1. Animals and Diets

Seven-week-old healthy male ApoE^−/−^ mice were procured from Vital River Laboratory Animal Technology (Beijing, China). The experimental subject in this study is an individual animal. Typically, there are 7 to 8 animals in each group. However, given that this experiment necessitates the assessment of aortic phenotypes and the sequencing of transcriptomes, adherence to the 3R principle led to the determination of 15 animals per group, totaling 45 animals for the entire study. Then, the mice were given adaptive feeding with a normal complete feed for 1 week to 8 weeks of age. Each animal was housed individually to prevent fighting, and feed consumption was recorded weekly from the start of the experimental treatment until the end of the experiment. The animals were numbered and randomly grouped using the random function of Excel and evenly distributed to treatment, control, and blank arms. For our experimental design, to overexpress *IGFBP5*, 40 μL of 1 × 10^12^ AAV-*IGFBP5*, 1 × 10^12^ AAV-GFP, or saline was injected intramuscularly into the leg skeletal muscle of ApoE^−/−^ mice once at 8 weeks of age. Adeno-associated virus was ordered from Hanheng Biotech (Shanghai, China). The vector used to construct the virus was pHBAAV-CMV-MCS-T2A-ZsGreen, which was ligated with the amplified CDS region of *IGFBP5* (NM_010518) using double restriction digestion, and the titer of the packaged virus was determined using quantitative PCR. Mice were maintained on a Western diet for 10 weeks. The feed was formulated specifically for the purpose of this study, consisting of 21% fat and 0.15% cholesterol, and was supplied by Vital River Company (Beijing, China).

Housing conditions included a climate-controlled environment set to a consistent 12-hour day-night cycle, with access to water and food provided ad libitum, keeping the conditions consistent between cages to minimize the interference of subtle environmental factors on the experiment. Euthanasia was carried out using an overdose of pentobarbital sodium (150 mg/kg body weight), administered via intraperitoneal injection at 20 weeks of age. Prior approval for all animal experiments was secured from the Xi’an Medical University Science and Technology Ethics Committee, Xi’an, China. All procedures were conducted in strict compliance with the institutional guidelines for animal welfare, as well as the ethical standards outlined in the “Guide for the Care and Use of Laboratory Animals” published by the United States National Institutes of Health, Bethesda, MD, USA (NIH publication number 85-23, revised 2011).

### 2.2. Serum Lipid Level Detection

Blood samples were collected from the mice through the tail vein at 0, 4, 8, and 12 weeks of high-fat feeding. To ensure accurate metabolic profiling, a 12-hour fasting period preceded each scheduled phlebotomy session. Harvested plasma specimens were promptly safeguarded at ultra-low temperatures (−80 °C) to preserve biochemical integrity. Quantitative assessments of total cholesterol (TC) levels were executed using commercially available assay kits provided by BioSino Bio-Technology & Science Inc., headquartered in Beijing, China. Similarly, determinations of high-density lipoprotein cholesterol (HDL-C) and low-density lipoprotein cholesterol (LDL-C) concentrations were obtained using diagnostic kits sourced from Nanjing Jiancheng Bioengineering Institute, also located in Nanjing, China. All analytical procedures were scrupulously executed in accordance with the manufacturers’ detailed protocols.

### 2.3. Quantification of Atherosclerotic Lesions

Termination of the mice was humanely obtained using an intraperitoneal administration of sodium pentobarbital at a concentration of 150 mg/kg. Subsequent dissection involved the careful excision and subsequent staining of the entire aortic tree utilizing Oil Red O. The saturated Oil Red O solution (G1015; Servicebio, Wuhan, China) was diluted with distilled water to 60% Oil Red O working solution, and the paraformaldehyde-fixed tissue was immersed for 30 min. This technique can specifically color neutral lipids such as triglycerides in cells or tissues, making the lipid deposition visible [21]. Further histological assessment entailed the preparation of frozen sections derived from the aortic root region. The upper part of the embedded heart was sliced serially. Then, 8 µm thick sections were collected starting from the time when the three aortic valve leaflets appeared simultaneously until the three aortic valve leaflets were incomplete in the section. Approximately 64 sections were collected for each sample. One section was selected every 7 sections, and a total of 8 sections were selected for subsequent staining statistics. All serial sections were stored at −80 °C until use. These sections underwent dual staining with Oil Red O to delineate areas of lipid accumulation and hematoxylin-eosin (H&E) to provide a comprehensive overview of tissue architecture, enabling quantitative measurement of lesion extent. For a detailed methodology and additional operational nuances, readers are encouraged to consult previous publications on the subject [21].

### 2.4. Western Blot Assay

The Western blotting procedure was conducted in accordance with established methodologies previously detailed [11]. The primary antibody used in this experiment was a rabbit anti-*IGFBP5* antibody (1:500, #55205-1-AP; Proteintech Group, Chicago, IL, USA). The secondary antibody used was horseradish peroxidase-conjugated goat anti-rabbit IgG (1:5000–1:10,000; GB23303; Servicebio, Wuhan, China).

### 2.5. RNA Isolation and RT-qPCR Analysis

Total RNA extraction was accomplished utilizing the RNAIso Plus reagent (Takara), followed by reverse transcription employing the PrimeScript^TM^ RT Reagent Kit (Takara), adhering to the protocol outlined in our preceding publication [11]. The primer sequences employed in this study are delineated in Table 1. Glyceraldehyde 3-phosphate dehydrogenase (GAPDH) served as an internal control for normalization purposes.

### 2.6. RNA-Seq Analysis

Total RNA isolation was obtained from the aortas of ApoE^−/−^ mice following treatment with either adeno-associated virus (AAV)-green fluorescent protein (GFP) or AAV-*IGFBP5*. Comprehensive transcriptome sequencing was executed by Panomix Biotech Co., Ltd. (Suzhou, Jiangsu, China). Differentially expressed genes (DEGs) were systematically characterized, applying stringent criteria involving a statistical threshold of *p* < 0.05 and a log2-fold change exceeding 1 in expression levels between the comparative groups.

### 2.7. Flow Cytometry Detection of Cell Cycle Distribution

To conduct cellular analysis, immortalized mouse aortic vascular smooth muscle cells (#IM-M108, IMMOCELL, Xiamen, China) were seeded at a subconfluent state of approximately 15–20% at passages 5–10. Upon reaching a density of 30–50%, the culture was divided into one untreated control and two treatment groups exposed to *IGFBP5* at concentrations of 150 ng/mL and 300 ng/mL, separately. Following a 24-hour exposure period, the cells underwent enzymatic dissociation using trypsin and were subsequently rinsed thrice with phosphate-buffered saline (PBS). After centrifugation at 1000 rpm for 5 min, the pelleted cells were suspended in 1 mL of 70% ethanol and maintained under refrigeration overnight for fixation. Subsequently, resuspended cells were washed again with PBS before being stained with 500 μL of propidium iodide (PI) staining solution, which contains 100 μg/mL PI, 1.0% Triton-X 100, and 2 mg/mL RNase in PBS. Incubation in darkness ensued for 30 min prior to flow cytometric analysis, targeting enumeration of 20,000–30,000 cells per sample. Each group in the experiment had three replicates, and the experiment was performed in triplicate.

### 2.8. CCK-8 Assay for Cell Proliferation

Cells were dispensed onto a 96-well microplate, and each well received 100 μL of Dulbecco’s modified Eagle medium (DMEM) enriched with 10% fetal bovine serum (FBS) for initiation of culture in 37 °C and 5% CO_2_. The experimental treatment is the same as described for the flow cytometry experiment in the previous paragraph. Following a 24-hour incubation, the growth medium was substituted with fresh DMEM inclusive of 10% Cell Counting Kit-8 (CCK-8) solution (C0038, Beyotime, Shanghai, China), initiating a secondary 3-hour incubation phase conducted under shielded conditions to preserve assay integrity. Upon completion, optical density readings were obtained at a spectral wavelength of 450 nanometers through spectrophotometric quantification using an enzyme-linked immunosorbent assay (ELISA) reader. Each group in the experiment had three replicates, and the experiment was performed in triplicate.

### 2.9. Cell Migration Assays

A scratch-wound healing assay was executed paralleling the procedures delineated in previous work [22]. Upon achieving monolayer confluence, cells were subjected to a controlled wounding procedure using a 200 μL pipette tip. Cells were washed 3 times with PBS to remove detached cells, and serum-free medium added. Images capturing the wound condition were documented at the immediate aftermath (0 h). Images capturing the wound condition were documented at 20-hour recovery interval after designated treatments. Each group in the experiment had three replicates, and the experiment was performed in triplicate.

### 2.10. Statistical Analysis

Statistical analysis was conducted on datasets represented as means accompanied by standard error margins (SEM), using Grubbs criterion, Dixon criterion, and *t*-test criterion to determine outliers, utilizing the robust capabilities of GraphPad Prism software, specifically version 9.00. To assess disparities amongst three or more groups demonstrating both equal variances and conformity to a Gaussian distribution, we implemented the one-way analysis of variance (ANOVA) test. Statistical significance was attributed to outcomes where *p*-values fell below the benchmark threshold of 0.05.

## 3. Results

### 3.1. Overexpression of IGFBP5 Does Not Affect Food Intake by and the Body Weight of ApoE^−/−^ Mice

**Figure 1:**
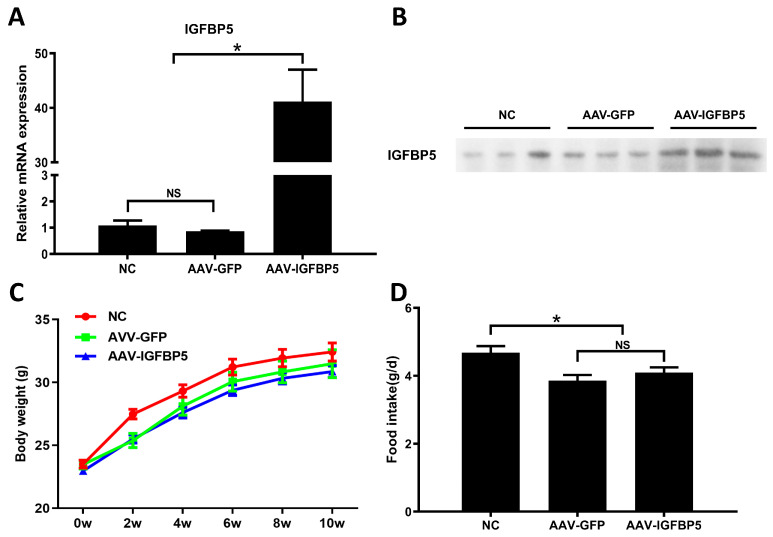
Overexpression of *IGFBP5* does not influence food consumption by and the body weight of ApoE^−/−^ mice. (**A**) *IGFBP5* mRNA expression level in skeletal muscle of ApoE^−/−^ mice at 10 weeks of experimental treatment (*n* = 3). (**B**) Plasma *IGFBP5* protein levels at 10 weeks of experimental treatment (*n* = 3). (**C**) Weight change curve (*n* = 15). (**D**) Comparison of average daily food intake during the entire experimental treatment among groups (*n* = 15). Data are expressed as the mean ± SEM. * *p* < 0.05. NS means no significant difference.

To induce overexpression of *IGFBP5*, adeno-associated viral vectors encoding *IGFBP5* were administered via direct injection into several sites across the hindlimbs of 8-week-old ApoE^−/−^ mice. Post-administration, quantitative real-time polymerase chain reaction (qRT-PCR) was deployed to ascertain the efficacy of *IGFBP5* expression within the skeletal muscle. Results indicated a remarkable augmentation in *IGFBP5* mRNA levels, achieving nearly a forty-fold increase relative to baseline measurements (Figure 1A). Furthermore, Western blot analyses revealed a pronounced elevation in the plasma concentration of *IGFBP5* protein, corroborating successful systemic delivery and expression (Figure 1B). Collectively, these findings underscore the feasibility of attaining enhanced circulating *IGFBP5* levels via localized muscular injection.

Upon 12 weeks of sustained exposure to a diet rich in saturated fats, comparative assessments of body mass and caloric consumption failed to reveal any discernible discrepancies between the *IGFBP5* overexpression cohort and respective controls. Thus, it appears that elevated *IGFBP5* does not exert appreciable influences on either overall weight gain or dietary intake patterns under conditions of chronic high-fat dietary challenge (Figure 1C,D).

### 3.2. Overexpression of IGFBP5 Increases Atherosclerotic Plaque Formation in ApoE^−/−^ Mice

**Figure 2:**
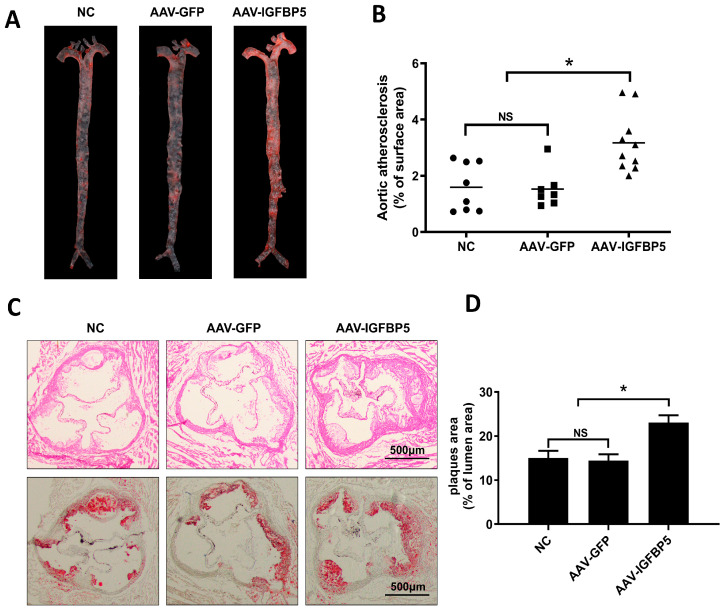
Overexpression of *IGFBP5* enhances the development of atherosclerotic plaques in ApoE^−/−^ mice. Oil Red O staining of the aorta (**A**) and statistical quantification (**B**). (**C**) H&E staining (up panel) and Oil Red O staining (down panel) of cardiac outflow tract sections. Scale bar: 500 μm. (**D**) Statistical quantification of neutral lipids in the plaques of the cardiac outflow tract sections stained with Oil Red O (*n* = 10). Data are expressed as the mean ± SEM. * *p* < 0.05. NS means no significant difference.

Upon conclusion of a 12-week regimen of high-fat feeding, ApoE^−/−^ mice underwent humane euthanasia. Post-sacrifice, the aortic trees were dissected and subsequently stained with Oil Red O—a specialized dye that selectively binds to lipid-laden plaques (Figure 2A). Analysis revealed that *IGFBP5* overexpression led to a marked escalation in the total surface area occupied by atheroma on the aortic walls, indicative of exacerbated atherosclerosis (Figure 2B).

Further exploration entailed the preparation of cross-sections from the cardiac outflow tract, which were then subjected to dual-staining protocols: hematoxylin-eosin (H&E) for general histological assessment and Oil Red O to accentuate lipid accumulation. Both staining methods collectively demonstrated that *IGFBP5* overexpression engendered a significant increase in plaque burden within the cardiac outflow tract, corroborating the notion that IGFBP5 plays a crucial role in the pathogenesis of atherosclerotic plaque formation in ApoE^−/−^ mice models (Figure 2C,D).

In summary, our experimental findings unequivocally highlight the capacity of *IGFBP5* overexpression to augment atherosclerotic plaque development in ApoE^−/−^ mice, implicating this protein as a potential therapeutic target for interventions aiming to curb cardiovascular diseases characterized by excessive plaque build-up.

### 3.3. Overexpression of IGFBP5 Has No Significant Effect on Blood Lipids in ApoE^−/−^ Mice

Given the pivotal role of blood lipids in the etiology of atherosclerotic plaque formation, we embarked on a longitudinal investigation to explore the potential mediating effects of *IGFBP5* on lipid profiles amidst a high-fat dietary regime. Mice were monitored and sampled at key timepoints—precisely at the onset of the study (0 weeks) and then at 4, 8, and 12 weeks post-initiation of the high-fat diet—to assess fluctuations in serum lipid concentrations.

**Figure 3:**
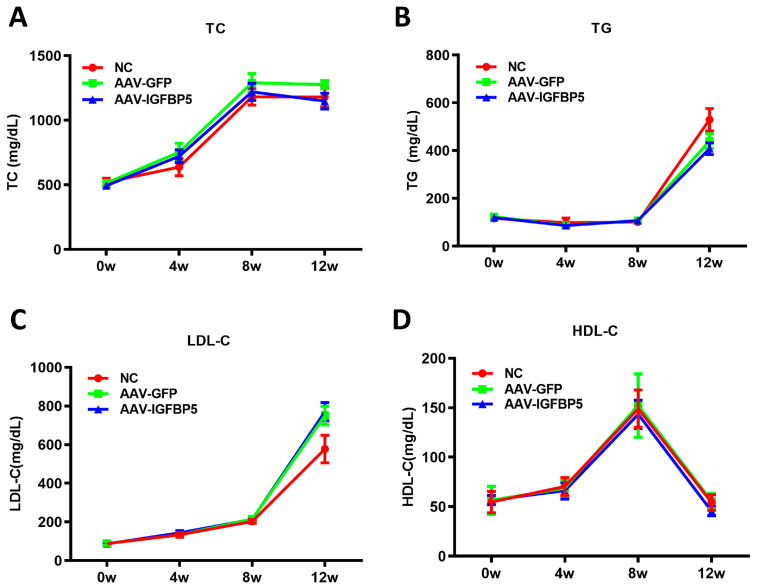
Effect of overexpression of *IGFBP5* on blood lipid levels in ApoE^−/−^ mice. Plasma was collected from each group of mice (*n* = 15) at week 0, 4, 8, and 12 of treatment to measure total cholesterol (TC) (**A**), total triglycerides (TG) (**B**), low-density lipoprotein cholesterol (LDL-C) (**C**), and high-density lipoprotein cholesterol (HDL-C) (**D**) levels. Data are expressed as the mean ± SEM.

Our meticulous biochemical assays revealed that while *IGFBP5* overexpression indeed induced alterations in atherogenesis, these effects did not manifest through significant shifts in traditional lipid markers. Specifically, total triglycerides (TG), total cholesterol (TC), low-density lipoprotein cholesterol (LDL-C), and high-density lipoprotein cholesterol (HDL-C) levels remained statistically indistinguishable between mice overexpressing *IGFBP5* and those harboring the empty vector (Figure 3). This observation suggests that the mechanism underlying *IGFBP5*-induced plaque augmentation diverges from conventional pathways associated with dyslipidemia.

### 3.4. Sequencing Analysis of Aortic Transcriptome of ApoE^−/−^ Mice After Overexpression of IGFBP5

Having ruled out the mediating role of blood lipids, we sought deeper insights into the molecular basis behind *IGFBP5*’s contribution to plaque escalation. To this end, we embarked on a comprehensive transcriptomic profiling endeavor focusing on the mouse aorta—the epicenter of atherosclerotic lesion formation. Employing next-generation sequencing technologies, we interrogated the global expression landscape of aortic tissues, subjecting them to rigorous bioinformatic scrutiny to identify genes with expression patterns that were significantly altered by *IGFBP5* overexpression.

**Figure 4:**
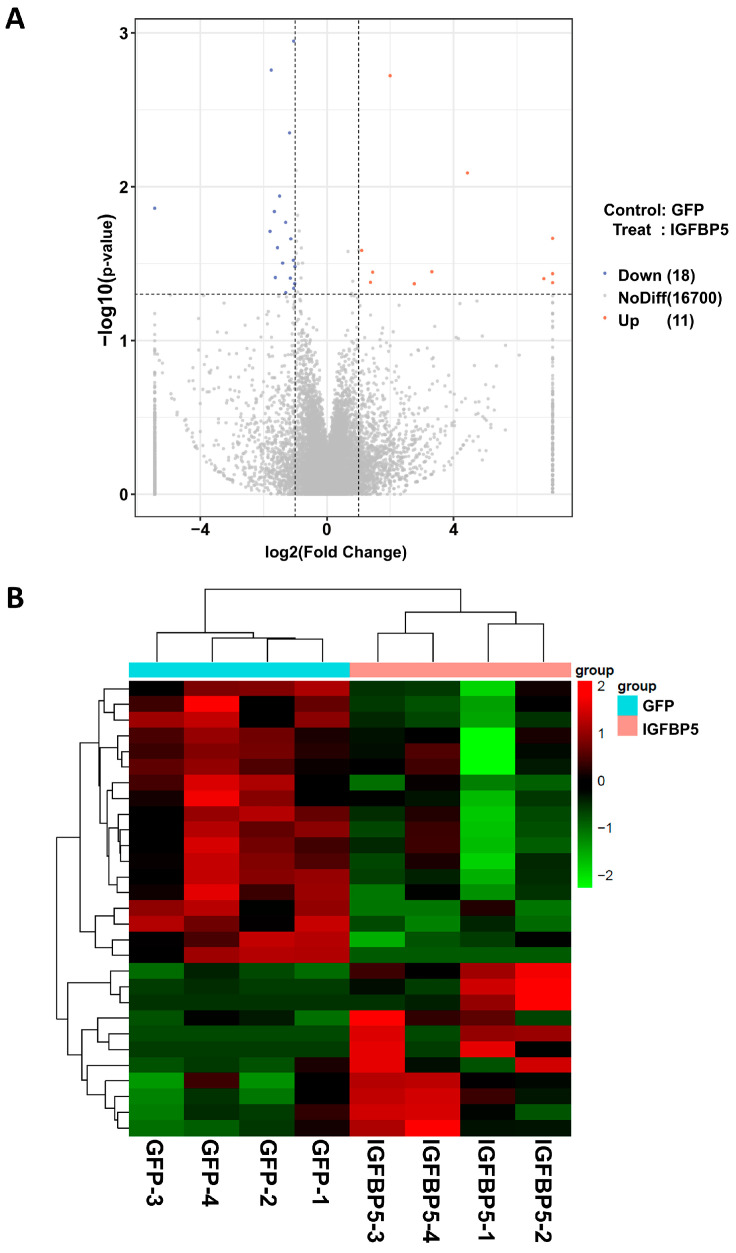
Transcriptome analysis of aortic tissues in ApoE^−/−^ mice following overexpression of *IGFBP5*. (**A**,**B**) Heatmap representing global gene expression in aortic tissues of AAV-GFP and AAV-*IGFBP5* mice after 12 weeks of being fed a HFD (*n* = 4).

This exhaustive analytical pipeline culminated in the identification of 16,729 genes and a sizable cohort comprising 29 differentially expressed genes (DEGs), partitioned into two subsets: 11 genes displayed enhanced expression (upregulated DEGs), whereas 18 genes exhibited reduced activity (downregulated DEGs) (Figure 4 and Supplementary Material File S1). The expression changes of some DEGs were verified by quantitative PCR, which was basically consistent with the sequencing results (Supplementary Figure S1). To contextualize these transcriptional changes within a functional framework, we performed Gene Ontology (GO) enrichment analysis on the identified DEGs.

**Figure 5:**
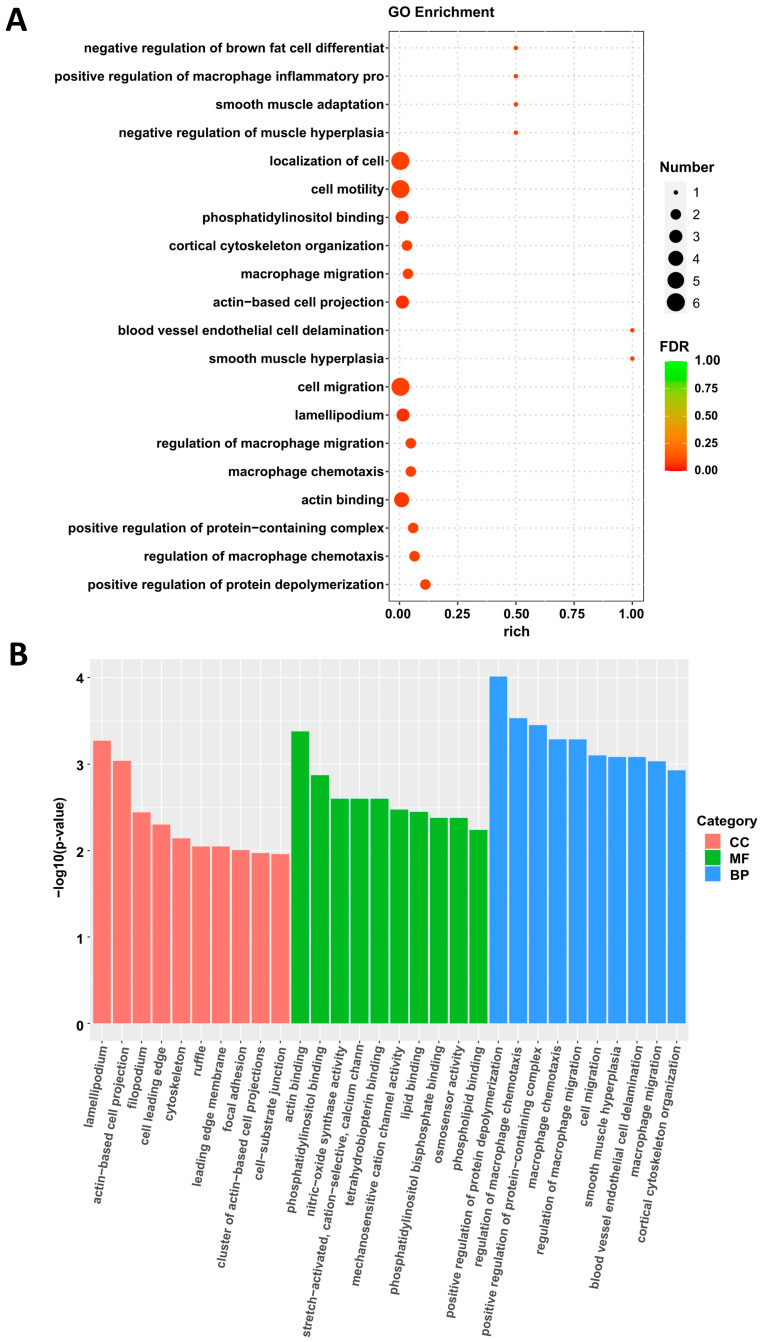
Gene Ontology analyses of the differentially expression genes in aortic tissues in ApoE^−/−^ mice following overexpression of *IGFBP5*. (**A**) Gene Ontology analyses of the differentially expression genes in aortic of AAV-GFP and AAV-*IGFBP5* mice after 12 weeks of HFD (*n* = 4). (**B**) GO analysis was performed based on three categories: biological process (BP), molecular function (MF), and cellular component (CC).

Results from the GO analysis illuminated a cluster of enriched biological processes, prominently featuring “smooth muscle cell proliferation” and “cell migration”—hallmarks pertinent to the pathobiology of atherosclerosis (Figure 5 and Supplementary Material File S2). This finding underscores how *IGFBP5*’s action on aortic cells could potentially alter plaque dynamics through modulating fundamental cellular activities rather than solely impacting lipid metabolism.

### 3.5. Overexpression of IGFBP5 Promotes the Transformation of VSMCs to a Proliferative Phenotype

**Figure 6:**
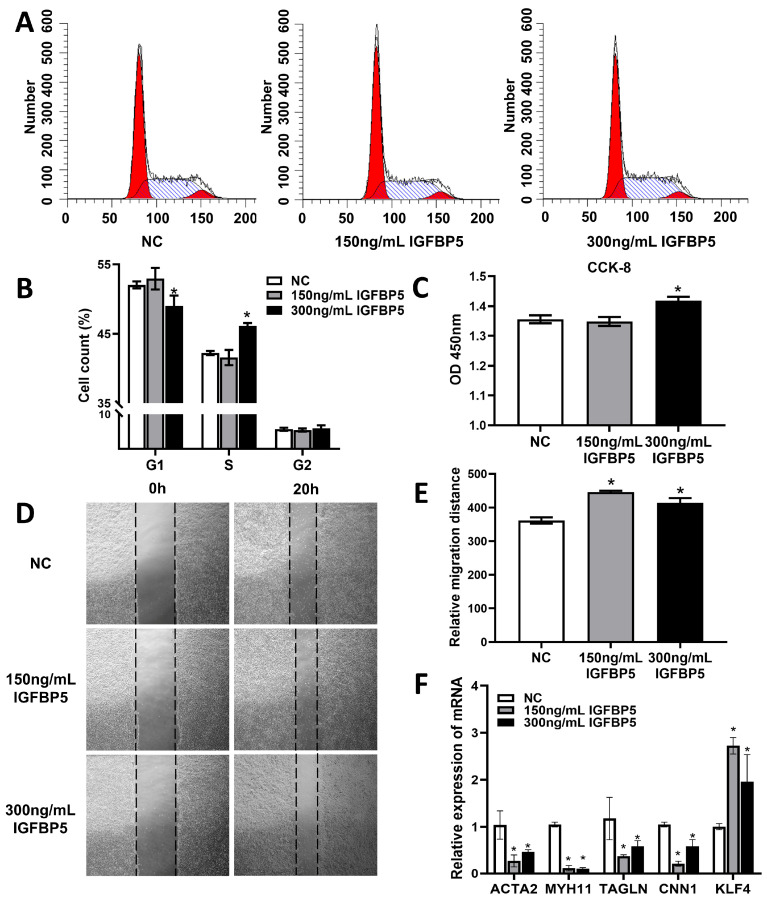
Overexpression of *IGFBP5* induces VSMCs to adopt a proliferative state. (**A**) Flow cytometric analysis of cell cycle distribution of immortalized mouse vascular smooth muscle cell line treated with different concentrations of *IGFBP5* during proliferation (*n* = 3). (**B**) Cell cycle distribution statistics (*n* = 3). (**C**) The CCK-8 kit was used to detect the effect of different concentrations of *IGFBP5* on VSMC proliferation for 24 h (*n* = 5). (**D**) Effects of different concentrations of *IGFBP5* on cell migration in cell scratch assays (*n* = 3). (**E**) Statistics of relative distance of cell migration (*n* = 3). (**F**) Real-time qPCR analysis of *ACTA2*, *MYH11*, *TAGLN*, *CNN1* and *KLF4* transcription expression in immortalized mouse vascular smooth muscle cells treated with different concentrations of *IGFBP5* (*n* = 3). *GAPDH* was used as a control. Data are expressed as the mean ± SEM. * *p* < 0.05.

Following our transcriptomic discoveries hinting at *IGFBP5*’s potential role in modulating VSMCs proliferation and migration, we proceeded to validate these observations using a series of complementary in vitro assays. Initially, employing flow cytometry, we assessed the cell cycle distribution in VSMCs treated with 300 ng/mL *IGFBP5*. Our findings revealed a significant decrease in the percentage of cells residing in the G1 phase, accompanied by a concomitant rise in the S phase fraction, suggesting that *IGFBP5* facilitates cell cycle progression, potentially contributing to enhanced VSMC proliferation (Figure 6A,B).

To quantify this proliferative effect, we utilized the Cell Counting Kit-8 (CCK-8) assay. Indeed, treatment with 300 ng/mL *IGFBP5* was shown to stimulate a robust increase in VSMC proliferation, further corroborating our earlier cell cycle analysis (Figure 6C).

Scratch wound healing assays provided additional evidence supporting *IGFBP5*’s stimulatory effect on VSMC migration. Both 150 ng/mL and 300 ng/mL doses of *IGFBP5* significantly accelerated closure rates compared to untreated controls, indicating a potent migratory response (Figure 6D,E).

To probe *IGFBP5*’s impact on VSMC phenotype, we turned to quantitative PCR (qPCR) to evaluate the expression of differentiation marker genes. Treatment with 150 ng/mL and 300 ng/mL *IGFBP5* consistently downregulated the mRNA levels of *ACTA2*, *MYH11*, *TAGLN*, and *CNN1*—key markers of VSMC differentiation—indicative of a dedifferentiated state conducive to proliferation and migration. Conversely, the mRNA expression level of Kruppel-like factor 4 (*KLF4*), a transcription factor essential for maintaining VSMC proliferation, was markedly upregulated in the IGFBP5-treated groups versus controls (Figure 6F), validating the phenotypic shift toward a less differentiated status.

## 4. Discussion

Atherosclerosis can precipitate a cascade of severe health complications, notably hypertension, coronary artery disease, angina pectoris, myocardial infarction (heart attack), and even sudden cardiac death [3,23]. The function of *IGFBP5* within the context of atherosclerosis remains a subject of considerable debate. To elucidate the exact role of *IGFBP5* in atherosclerosis, we overexpressed *IGFBP5* in the classic atherosclerosis mouse model, ApoE^−/−^ mice. The resultant data unequivocally suggested that *IGFBP5* exacerbates atherosclerosis in these models, manifesting primarily as accelerated plaque formation. Remarkably, despite its potent atherogenic effects, *IGFBP5* overexpression appeared to spare the lipid profiles of the mice, indicating that its influence is not caused by blood lipids. As mentioned earlier, Xu et al.’s work showed that *IGFBP5* inhibits monocyte adhesion to endothelial cells and reduces endothelial cell inflammation [20]. In addition, the latest studies have shown that *IGFBP5* negatively regulates the M1 polarization of monocytes/macrophages and inhibits the inflammatory response of monocytes/macrophages [24]. This dichotomy raises questions regarding the primary conduit through which *IGFBP5* catalyzes plaque deposition. It is plausible that the principal mechanism underlying *IGFBP5*’s promotion of atherosclerotic lesion development lies in its modulation of vascular smooth muscle physiology. To further probe this hypothesis, transcriptomic analysis was conducted on the aortas of mice subjected to *IGFBP5* overexpression, unveiling striking alterations in gene expression patterns pertinent to vascular smooth muscle proliferation and migratory capabilities. Consequently, our current endeavor seeks to rigorously validate *IGFBP5*’s purported effects on vascular smooth muscle biology, aiming to decipher whether its impact indeed pivots around these crucial cellular functions.

The outcomes of our recent experimentation elucidate a pivotal function of *IGFBP5* in enhancing the proliferation and migratory capacity of VSMCs. These findings resonate with prior investigations by Yu et al., whose studies on the rat A7R5 smooth muscle cell line confirmed that *IGFBP5* acts as a critical intermediary in Angiotensin II (AngII)-stimulated proliferation and movement of VSMCs [25]. Such corroboration reinforces the significance of *IGFBP5* in cardiovascular pathophysiology. In addition, the mTOR pathway has been confirmed by many studies to be involved in VSMC proliferation and migration [26,27]. Studies have found that *miR-137* inhibited cell proliferation and migration of VSMCs via targeting *IGFBP5* and modulating the *mTOR/STAT3* signaling [28]. Therefore, *IGFBP5* may regulate VSMC proliferation and migration through the *mTOR* pathway.

Moreover, our comprehensive analyses revealed a concurrent decrease in the expression of contractile marker genes specific to VSMCs. This downregulation is indicative of a shift from a contractile to a synthetic phenotype, often associated with increased cellular proliferation. This observation not only corroborates the existing literature but also underscores the multifaceted effects of *IGFBP5* on VSMC biology.

The anti-inflammatory effect of *IGFBP5* proposed by Xu et al. is not an isolated case. The latest research showing that *IGFBP5* negatively regulates M1 polarization of monocytes/macrophages. *IGFBP5* silencing led to increased secretion of *IL-1β* and *IL-6*. At the same time, the study also found that in the acute inflammatory model, the levels of *IGFBP5* in leukocytes and serum decreased, but in chronic inflammatory diseases, the expression of *IGFBP5* in leukocytes decreased, but the level of *IGFBP5* in serum increased significantly. These excessive levels of *IGFBP5* mainly come from white adipose tissue, and the excessive levels of *IGFBP5* in serum interfere with the anti-inflammatory effect of *IGF1* on monocytes, which actually causes a pro-inflammatory phenotype [24]. Atherosclerosis itself is a chronic inflammatory disease. This study achieved experimental treatment by overexpressing *IGFBP5* in skeletal muscle to increase serum *IGFBP5* levels and maintained it for a long time. The study by Xu et al. was a short-term local study conducted based on overexpression in endothelial cells. The contradiction between the two is similar to the study by Fan et al., so further research is needed in this regard.

Several studies indicate that the increase in serum *IGFBP5* levels increases the risk of cardiovascular disease [14,15,16], but it is not clear how serum *IGFBP5* causes the disease. This study confirmed that the increase in serum *IGFBP5* levels would aggravate the formation of atherosclerosis through research on the cardiovascular pathology-based atherosclerosis model and found that the main cause of the phenotype focused on vascular smooth muscle, rather than abnormal lipid metabolism. Its mechanism of action involves stimulating VSMC proliferation, facilitating their migration, and inducing a phenotypic transition conducive to plaque instability. These findings highlight *IGFBP5* as a potential therapeutic target for mitigating cardiovascular diseases characterized by atherosclerosis. Of course, the results of this study are limited to male ApoE^−/−^ mice and immortalized mouse aortic vascular smooth muscle cells, and these results need to be further confirmed in other animal models and human samples.

## 5. Conclusions

*IGFBP5* promotes atherosclerosis in ApoE^−/−^ mice through the phenotypic transformation of VSMCs. This finding suggests that *IGFBP5* has the potential to serve as an indicator of atherosclerosis diagnosis and a target for therapeutic interventions in the future.

## Figures and Tables

**Table 1:** Primers used in the present study.

Names	Sequence
*IGFBP5*	F: AGATGAGACAGGAATCCGAACAAG R: GAAGGCGTGGCACTGAAAG
*ACAT2*	F: GTCCCAGACATCAGGGAGTAA R: TCGGATACTTCAGCGTCAGGA
*MYH11*	F: AAGCTGCGGCTAGAGGTCA R: CCCTCCCTTTGATGGCTGAG
*TAGLN*	F: CAACAAGGGTCCATCCTACGG R: ATCTGGGCGGCCTACATCA
*CNN1*	F: TCTGCACATTTTAACCGAGGTC R: GCCAGCTTGTTCTTTACTTCAGC
*KLF4*	F: GTGCCCCGACTAACCGTTG R: GTCGTTGAACTCCTCGGTCT
*GAPDH*	F: TCGCTCCTGGAAGATGGTGAT R: CAGTGGCAAAGTGGAGATTGTTG

## Data Availability

The data that support the findings of this study are available from the corresponding author upon reasonable request.

## Supplementary Materials

The following supporting information can be downloaded at: https://www.mdpi.com/article/10.3390/cimb47070555/s1.

## Abbreviations

The following abbreviations are used in this manuscript:

VSMCs	Vascular smooth muscle cells
IGFBP5	Insulin-like growth factor binding protein 5
ApoE	Apolipoprotein E
GO	Gene Ontology
AAV	Adeno-associated virus
TC	Total cholesterol
TG	Total triglyceride
HDL-C	High-density lipoprotein cholesterol
LDL-C	Low-density lipoprotein cholesterol
DEGs	Differentially expressed genes
H&E	Hematoxylin-eosin
GFP	Green fluorescent protein
CCK-8	Cell counting kit-8
DMEM	Dulbecco’s modified Eagle medium
FBS	Fetal bovine serum
GAPDH	Glyceraldehyde 3-phosphate dehydrogenase
PI	Propidium iodide
ACTA2	Actin alpha 2
MYH11	Myosin heavy chain 11
TAGLN	Transgelin
CNN1	Calponin 1
KLF4	Kruppel-like factor 4

## References

[1] Roth G.A., Mensah G.A., Johnson C.O., Addolorato G., Ammirati E., Baddour L.M., Barengo N.C., Beaton A.Z., Benjamin E.J., Benziger C.P. (2020). Global Burden of Cardiovascular Diseases and Risk Factors, 1990–2019: Update From the GBD 2019 Study. J. Am. Coll. Cardiol..

[2] Soehnlein O., Libby P. (2021). Targeting inflammation in atherosclerosis-from experimental insights to the clinic. Nat. Rev. Drug Discov..

[3] Libby P., Buring J.E., Badimon L., Hansson G.K., Deanfield J., Bittencourt M.S., Tokgozoglu L., Lewis E.F. (2019). Atherosclerosis. Nat. Rev. Dis. Primers.

[4] Gimbrone M.A., Garcia-Cardena G. (2016). Endothelial Cell Dysfunction and the Pathobiology of Atherosclerosis. Circ. Res..

[5] Libby P. (2021). The changing landscape of atherosclerosis. Nature.

[6] Cao G., Xuan X., Hu J., Zhang R., Jin H., Dong H. (2022). How vascular smooth muscle cell phenotype switching contributes to vascular disease. Cell Commun. Signal..

[7] Yu Y., Cai Y., Yang F., Yang Y., Cui Z., Shi D., Bai R. (2024). Vascular smooth muscle cell phenotypic switching in atherosclerosis. Heliyon.

[8] Duan C., Allard J.B. (2020). Insulin-Like Growth Factor Binding Protein-5 in Physiology and Disease. Front. Endocrinol..

[9] Bach L.A. (2015). Insulin-like growth factor binding proteins 4–6. Best Pract. Res. Clin. Endocrinol. Metab..

[10] Xiao Z., Chu Y., Qin W. (2020). IGFBP5 modulates lipid metabolism and insulin sensitivity through activating AMPK pathway in non-alcoholic fatty liver disease. Life Sci..

[11] Xiang A., Chu G., Zhu Y., Ma G., Yang G., Sun S. (2019). IGFBP5 suppresses oleate-induced intramyocellular lipids deposition and enhances insulin signaling. J. Cell. Physiol..

[12] Hsieh T., Gordon R.E., Clemmons D.R., Busby W.H., Duan C. (2003). Regulation of vascular smooth muscle cell responses to insulin-like growth factor (IGF)-I by local IGF-binding proteins. J. Biol. Chem..

[13] Kuemmerle J.F., Zhou H. (2002). Insulin-like growth factor-binding protein-5 (IGFBP-5) stimulates growth and IGF-I secretion in human intestinal smooth muscle by Ras-dependent activation of p38 MAP kinase and Erk1/2 pathways. J. Biol. Chem..

[14] Fischer F., Schulte H., Mohan S., Tataru M.C., Kohler E., Assmann G., von Eckardstein A. (2004). Associations of insulin-like growth factors, insulin-like growth factor binding proteins and acid-labile subunit with coronary heart disease. Clin. Endocrinol..

[15] Wang Q., Chi J., Zeng W., Xu F., Li X., Wang Z., Qu M. (2024). Discovery of crucial cytokines associated with deep vein thrombus formation by protein array analysis. BMC Cardiovasc. Disord..

[16] Zhu Q., Cheang I., Guo Q., Lu X., Li Y., Yao W., Zhang H., Li X. (2024). Serum IGFBP5 as a predictor of major adverse cardiac events in patients with acute myocardial infarction. Int. J. Cardiol..

[17] Zheng B., Duan C., Clemmons D.R. (1998). The effect of extracellular matrix proteins on porcine smooth muscle cell insulin-like growth factor (IGF) binding protein-5 synthesis and responsiveness to IGF-I. J. Biol. Chem..

[18] Kim K.S., Seu Y.B., Baek S.H., Kim M.J., Kim K.J., Kim J.H., Kim J.R. (2007). Induction of cellular senescence by insulin-like growth factor binding protein-5 through a p53-dependent mechanism. Mol. Biol. Cell.

[19] Sanada F., Muratsu J., Otsu R., Shimizu H., Koibuchi N., Uchida K., Taniyama Y., Yoshimura S., Rakugi H., Morishita R. (2017). Local Production of Activated Factor X in Atherosclerotic Plaque Induced Vascular Smooth Muscle Cell Senescence. Sci. Rep..

[20] Xu S., Xu Y., Yin M., Zhang S., Liu P., Koroleva M., Si S., Little P.J., Pelisek J., Jin Z.G. (2018). Flow-dependent epigenetic regulation of IGFBP5 expression by H3K27me3 contributes to endothelial anti-inflammatory effects. Theranostics.

[21] Guan H., Liu T., Liu M., Wang X., Shi T., Guo F. (2023). SFRP4 Reduces Atherosclerosis Plaque Formation in ApoE Deficient Mice. Cardiol. Res. Pract..

[22] Su P., Tian Y., Yin C., Wang X., Li D., Yang C., Pei J., Deng X., King S., Li Y. (2022). MACF1 promotes osteoblastic cell migration by regulating MAP1B through the GSK3beta/TCF7 pathway. Bone.

[23] Mlynarska E., Czarnik W., Fularski P., Hajdys J., Majchrowicz G., Stabrawa M., Rysz J., Franczyk B. (2024). From Atherosclerotic Plaque to Myocardial Infarction-The Leading Cause of Coronary Artery Occlusion. Int. J. Mol. Sci..

[24] Fan Y., Wu Y.J., Guo K., Zhou X.Q., Abulaiti A., Olatunji O.J., Ji C.L., Zuo J. (2024). Interaction with IGF1 overrides ANXA2-mediated anti-inflammatory functions of IGFBP5 in vivo. Front. Immunol..

[25] Ha Y.M., Nam J.O., Kang Y.J. (2015). Pitavastatin Regulates Ang II Induced Proliferation and Migration via IGFBP-5 in VSMC. Korean J. Physiol. Pharmacol..

[26] Lu Q.B., Wan M.Y., Wang P.Y., Zhang C.X., Xu D.Y., Liao X., Sun H.J. (2018). Chicoric acid prevents PDGF-BB-induced VSMC dedifferentiation, proliferation and migration by suppressing ROS/NFkappaB/mTOR/P70S6K signaling cascade. Redox Biol..

[27] Zhang M., Li F., Wang X., Gong J., Xian Y., Wang G., Zheng Z., Shang C., Wang B., He Y. (2020). MiR-145 alleviates Hcy-induced VSMC proliferation, migration, and phenotypic switch through repression of the PI3K/Akt/mTOR pathway. Histochem. Cell Biol..

[28] Pan J., Li K., Huang W., Zhang X. (2017). MiR-137 inhibited cell proliferation and migration of vascular smooth muscle cells via targeting IGFBP-5 and modulating the mTOR/STAT3 signaling. PLoS ONE.

