# Supplementary material for: IGFBP5 Promotes Atherosclerosis in APOE−/− Mice Through Phenotypic Transformation of VSMCs
Source: Curr Issues Mol Biol. 2025 Jul 17;47(7):555. doi: 10.3390/cimb47070555 (PMC12293107; doi:10.3390/cimb47070555)
Supplement: Supplementary file 1 [file cimb-47-00555-s001.zip › supplementary figure S1.pdf]

Supplementary Figure S1

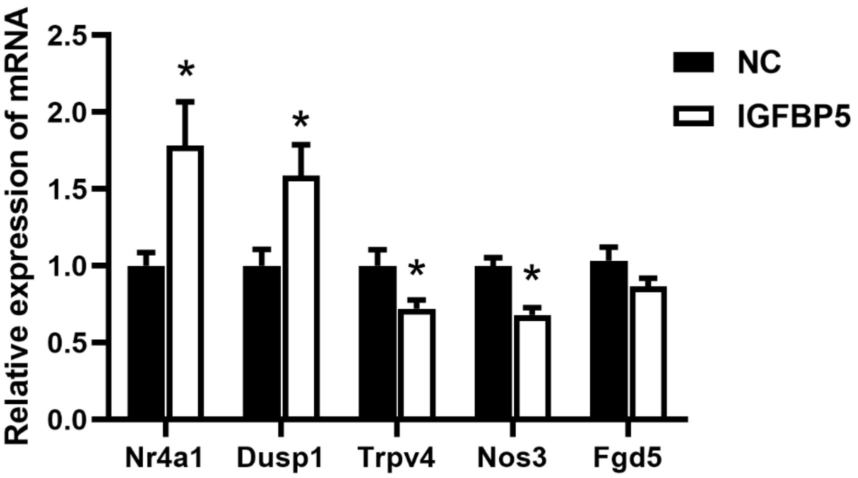

Supplementary Figure S1. Quantitative PCR validation of transcriptome sequencing results. Nuclear receptor subfamily 4 group A member 1（Nr4a1）、Dual-specificity phosphatase 1（Dusp1）、Transient receptor potential vanilloid 4（Trpv4）、Nitric oxide synthase 3（Nos3）、and Faciogenital dysplasia 5（Fgd5）were detected respectively (n = 3).
